# Adult-onset Still’s disease with multiple lymphadenopathy: a case report and literature review

**DOI:** 10.1186/s13000-021-01159-3

**Published:** 2021-10-27

**Authors:** Zhonghua Huang, Hua Xu, Qinqin Min, Zhenguo Li, Jiaxin Bi, Lingyun Liu, Yingying Liang

**Affiliations:** Department of Pathology, Shenzhen Traditional Chinese Medicine Hospital, The Fourth Clinical Medical College of Guangzhou University of Chinese Medicine, Shenzhen, 518033 Guangdong Province China

**Keywords:** Adult-onset Still’s disease, Lymphadenopathy, Lupus lymphadenitis, Autoinflammatory diseases, Case report

## Abstract

**Background:**

Adult-onset Still’s disease (AOSD) often presents with systemic multiple lymphadenopathy. In addition to the common paracortical and mixed patterns in AOSD lymph node histopathological features, other morphological patterns include diffuse, necrotic, and follicular patterns. However, to date, there have been few reports on the histopathological description of AOSD lymph nodes.

**Case presentation:**

An 18-year-old woman presented 2 months earlier with pain in her large joints with painless rash formation; bilateral posterior cervical lymph node, left supraclavicular lymph node, and left posterior axillary lymph node enlargement, and no tenderness. Left cervical lymph node resection was performed for pathological examination. The lymph node structure was basically preserved, and subcapsular and medullary sinus structures were observed. Many histiocytes in the sinus were observed, the cortical area was reduced, a few lymphoid follicles of different sizes were observed, and some atrophy and hyperplasia were noted. The lymphoid tissue in the paracortical region of the lymph node was diffusely proliferative and enlarged, mainly comprising histiocytes with abundant cytoplasm, immunoblasts and numerous lymphocytes with slightly irregular, small- to medium-sized nuclei. Nuclear karyorrhexis was easily observed, showing a few nuclear debris and the “starry sky” phenomenon, accompanied by abundantly branching high endothelial small vessels with few scattered plasma cells and eosinophil infiltration. Lymphoid follicle immunophenotype with reactive proliferative changes was observed. Approximately 40% of the cells in the paracortical region were positive for Ki-67, and the histiocytes expressed CD68, CD163, and some expressed S-100, with the absence of myeloperoxidase. The immunoblasts expressed CD30 and CD20, not ALK or CD15. Background small- to medium-sized T cells expressed CD2, CD3, CD5, CD7, CD4, and CD8; the number of CD8-positive T cells was slightly predominant, and a small number of T cells expressed granzyme B and T-cell intracellular antigen 1. The patient received a comprehensive medical treatment after the operation, and her condition was stable without progression at the 11-month follow-up evaluation.

**Conclusions:**

The pathological features of AOSD lymphadenopathy raises the awareness of AOSD among pathologists and clinicians and aids in the diagnosis and differential diagnosis of AOSD lymphadenopathy from other reactive lymphadenopathies (lupus lymphadenitis, etc.) and lymphomas.

## Background

Adult-onset Still’s disease (AOSD) is a rare group of systemic autoinflammatory diseases with complex, incompletely defined etiology and pathogenesis, mainly characterized by intermittent hyperthermia, transient skin rash, elevated blood leukocyte levels (neutrophils > 80%), polyarthritic pain, and multiple lymphadenopathy, and a predilection for young adults [1]. In 1971, Bywaters first described 14 cases of Still’s disease observed in patients aged 17–35 years with clinical features significantly similar to those of childhood Still’s disease, mainly characterized by high fever, multiple skin rashes, and polyarthritis, thus defining AOSD [2]. AOSD is often accompanied by liver and spleen enlargement and lymphadenopathy. Its clinical manifestations are complex and unspecific, sometimes similar and overlapping with lymphoma in clinical manifestations and histopathology [3], which may easily lead to misdiagnosis or missed diagnosis. In this case report, we present a case of AOSD and review the relevant literature to explore the clinical and pathomorphological features of enlarged lymph nodes and immunophenotypes, with the aim of improving the level of pathological diagnosis of the disease to prevent the misdiagnosis of lymphoma or other associated lymphadenopathies (lupus lymphadenitis, etc.).

## Case presentation

**Fig. 1:**
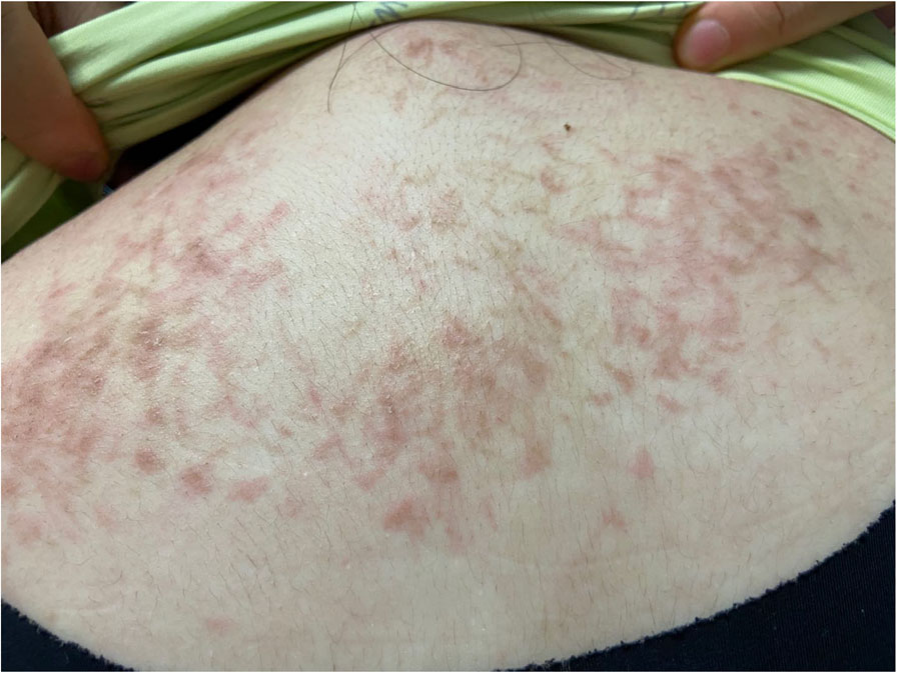
Pruritus and painless rash developed on the back and shoulder skin of the patient

Our patient was an 18-year-old woman. Two months earlier, she presented with pain in the large joints of all extremities, with no evident trigger for its development, and pruritic and painless rash formation on the skin of the dorsum of the shoulders (Fig. 1) and both wrists and both sides of the thighs, which worsened with increasing symptoms. Febrile and night sweats were observed on the sixth day upon admission. The patient was admitted to the hospital for blood analysis, and the blood test results were as follows: ferritin, 466.4 ng/ml; C-reactive protein (CRP) level, 82.7 mg/l; interleukin-6 level, 85.12 pg/ml; white blood cell count, 15.55 × 10^9^/L; and neutrophils, 89.3%. The patient’s antinuclear antibody was positive (titer 1:100), but her rheumatoid factor,anti double stranded DNA antibody, Anti smooth muscle antibody,anti-extractable nuclear antigen antibodies and anti-neutrophil cytoplasm antibodies were negative. Since symptom onset, the patient lost 5 kg of body weight. Examination revealed the presence of bilateral posterior cervical lymph nodes, left supraclavicular lymph nodes, and left posterior axillary lymph nodes without tenderness. According to the new classification of systemic lupus erythematosus (SLE) published by the European alliance against Rheumatism (EULAR) and the American Society of Rheumatology (ACR) in 2019 [4], clinicians have excluded the diagnosis of SLE. Following the clinical suspicion of lymphoma, left neck lymph node resection was performed for pathological examination.

### Pathological findings

One lymph node was resected, measuring approximately 2.5 × 2 × 1.2 cm, with its cut surface gray white and gray red in color and medium in texture.

**Fig. 2:**
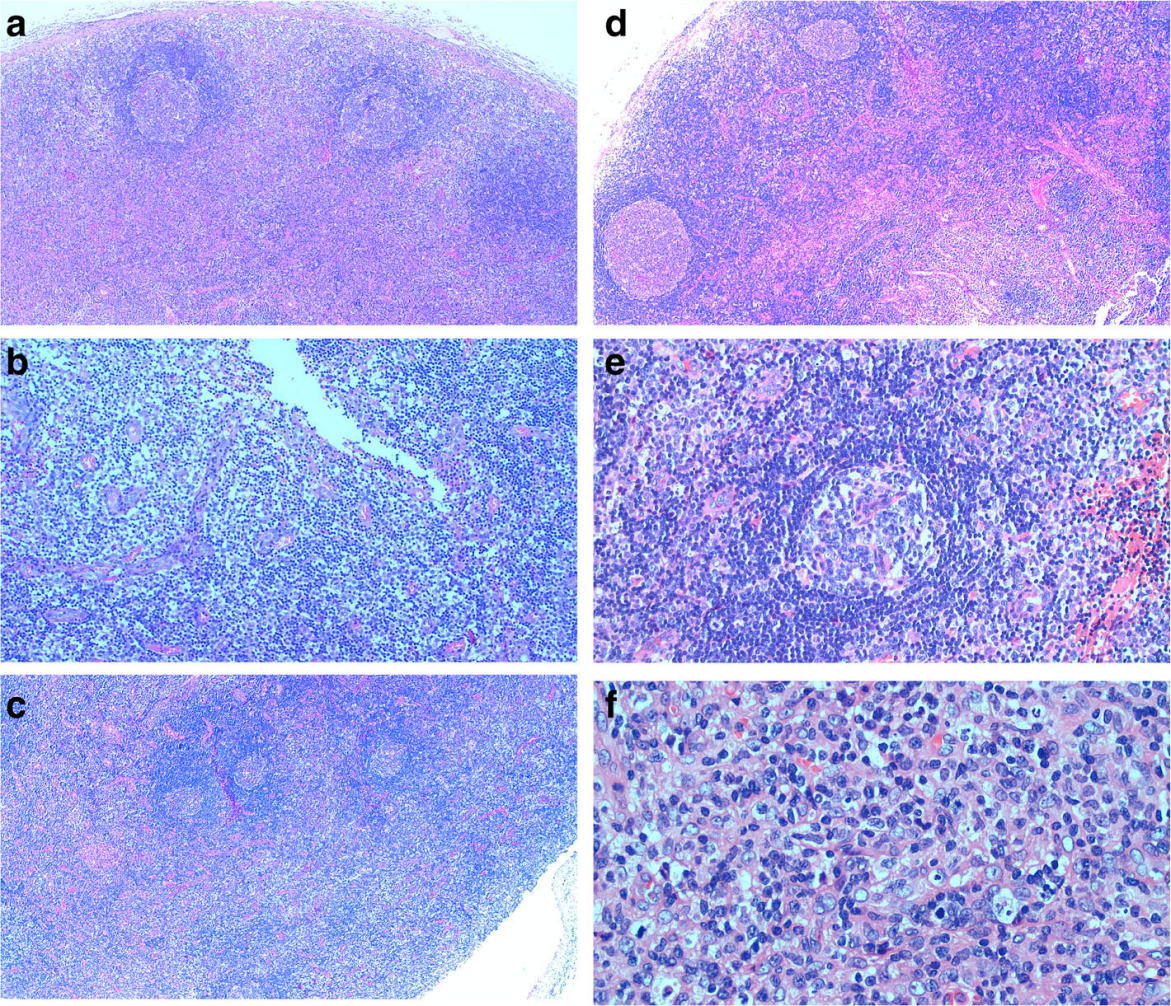
Microscopic features. **a**. Lymph node structure is partially preserved, and residual lymph follicles can be seen (HE, × 40);**b**. The subcapsular and medullary sinuses are slightly expanded, and the sinus comprises a larger amount of histiocytes (HE, × 100);**c**. The cortical areas are atrophic and smaller (HE, × 40);**d**. A few lymphoid follicles of various sizes are observed (HE, × 40);**e**. Some of the follicular germinal centers are atrophic and smaller (HE, × 100);f. Lymph node paracortical areas are diffusely proliferative and enlarged,and consist mainly of histiocytes, immunoblasts, and medium-sized T lymphocytes (HE, × 400)

**Fig. 3:**
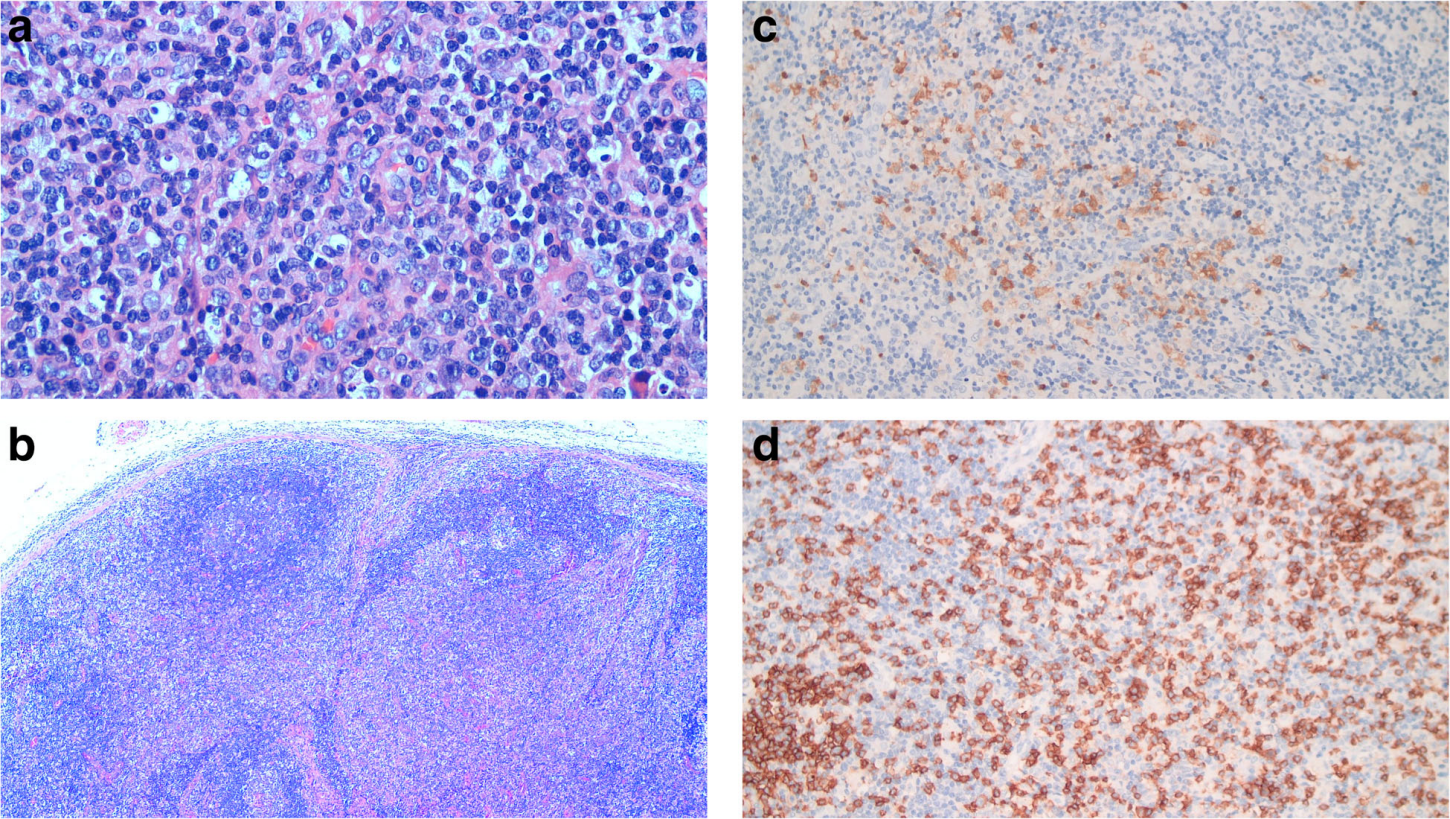
Microscopic features. **a**. A small number of histiocytes (black arrows) have distorted and elongated nuclei with irregular shapes (HE, × 400);**b**. High endothelial venule proliferation in the paracortical area (HE, × 40);**c**. The proliferating histiocytic fraction of the paracortical area expresses S-100(Envison, × 100);**d**. Proliferating CD8-positive T cells in the paracortical zone (Envison, × 100)

The lymph node structure was partially preserved (Fig. 2a),the subcapsular and medullary sinuses were slightly dilated, and the sinus comprised a larger amount of histiocytes (Fig. 2b). The cortical areas were atrophic and smaller (Fig. 2c), and a few lymphoid follicles of various sizes were observed (Fig. 2d). Some of the follicular germinal centers were atrophic and smaller (Fig. 2e), some follicles germinal centers were hyperplastic and enlarged with the phenomenon of “starry sky”.The paracortical areas of the lymph nodes were diffusely hyperplastic and enlarged,mainly comprised abundant histiocytes, immunoblasts and medium-sized T lymphocytes with slightly irregular nuclei,against a background scattered with a higher amount of plasma cells and a few infiltrating eosinophils (Fig. 2f). In some areas, histiocytic hyperplasia was patchy,and a few proliferating histiocytes had distorted and elongated nuclei with irregular morphology (Fig. 3a). In some areas, immunoblasts proliferated in a “mottled” manner, with a small amount of apoptotic nuclear debris and phagocytosis of nuclear debris by histiocytes. Medium-sized T lymphocytes were actively proliferative,karyorrhexis was easily observed, and the proliferation of high endothelial venules in the paracortical area showed a complex branching pattern (Fig. 3b).

### Immunohistochemical findings

Proliferating histiocytes expressed CD68 and CD163, and some expressed S-100 (Fig. 3c) and myeloperoxidase. The proliferating immunoblasts were strongly and weakly heterogeneously positive for CD30 and CD20,and negative for CD15.The proliferating medium-sized T lymphocytes in the paracortical areas expressed CD3, CD5, CD4, and CD8, with a slight predominance of CD8-positive cells (Fig. 3d), and some T cells expressed granzyme B and T-cell intracellular antigen 1. The proliferation index of Ki-67 in the hot spots of paracortical areas was about 40%, and plasma cells expressed CD138 but not immunoglobulin 4(IgG4). Lymphoid follicles expressed CD20, CD10, and BCL-6,and did not express BCL-2,the proliferation index of Ki-67 was approximately 80%. CD123, CD56, TdT, ALK, and CK antibodies were not detected.

The final diagnosis was adult-onset Still’s disease (AOSD), combined with clinical manifestations, laboratory examination and lymph node pathological biopsy. The patient received a comprehensive medical treatment after the operation, including symptomatic treatment, oral prednisone acetate tablets and methotrexate. Her condition was stable without progression at the 11-month follow-up evaluation.

## Discussion and conclusions

Clinically, AOSD is relatively rare, with an incidence between approximately 1 and 34 per 1 million population, with an equal incidence in both sexes, and a “bimodal” age of onset of 15–25 years and 36–46 years [5]. AOSD often has four major clinical features: transient rash in the proximal limbs or trunk at the peak of fever, high fever of 39 °C or more, elevated peripheral white blood cell count and neutrophil proportion greater than 80%, and generalized polyarticular pain or arthritis. Other clinical manifestations include pharyngeal pain, myalgia, myositis, lymphadenopathy, splenomegaly, pericarditis, myocarditis, pleuritis, lung disease, hepatitis, increased erythrocyte sedimentation rate and CRP levels, increased ferritin level, decreased glycosylated ferritin level, and coagulopathy [6]. AOSD can be easily misdiagnosed clinically as infectious lesions or other autoimmune diseases,such as systemic lupus erythematosus,because patients with systemic lupus erythematosus often have joint pain and rash. Clinicians are not aware of the possibility of AOSD when conservative treatment fails. After futile antibiotic treatment such as in the case presented here, AOSD was finally diagnosed by pathological examination of biopsied lymph nodes. Studies have shown that the general time from the appearance of symptoms or signs to the final diagnosis of AOSD ranges from 1.5 to 4 years [7].

AOSD usually presents with high fever, arthralgia, and rash and is often accompanied by multiple lymphadenopathy and hepatosplenomegaly [8]. When malignant lymphoma is easily suspected clinically after conservative medical treatment is ineffective, pathological biopsy of lymph nodes is the inevitable choice, combined with pathomorphological features, immunohistochemistry, and molecular biological examination to confirm the diagnosis. Jeon et al. [9] have summarized the pathohistomorphological changes in 12 AOSD enlarged lymph nodes and classified the lymphadenopathy into four morphological types. The first atypical paracortical hyperplasia pattern, characterized by hyperplasia in the paracortical areas of the lymph nodes with abundant high endothelial vessels, comprised mainly of reactive proliferating T lymphocytes, scattered large activated B/T immunoblasts, and few plasma cells and eosinophils, with a ratio of CD4- to CD8-positive T lymphocytes of approximately 3:2. Moreover, mildly hyperplastic histiocytes and focally hyperplastic monocytoid B cells were observed. Furthermore, the second burnt out histiocytic pattern was characterized by hyperplasia of the paracortical areas, high endothelial vascularity and sinus histiocytic proliferation with no remnants of lymphoid follicles. Histiocytes expressed CD68 and S-100 and were often clustered in a mottled pattern in the paracortical areas. Additionally, the third exuberant immunoblastic reaction pattern was characterized by patchy or diffuse proliferation of numerous immunoblasts in the paracortical area, predominant immunoblasts with numerous mitotic figures and a Ki-67 proliferation index of up to 90%, which is most easily confused with malignant lymphoma. The fourth follicular hyperplasia pattern was characterized by numerous lymphoid follicles of various sizes distributed throughout the lymph nodes, some with enlarged germinal centers, some with atrophy of germinal centers, and vascular hyalinization with widening of the mantle or marginal zones. The histomorphological features of AOSD lymphadenopathy are complex and diverse and change dynamically with the course of the disease.

Kim et al. [10] have performed histological observation of lymphadenopathy in 48 patients with AOSD and summarized six morphological patterns of lymph nodes. These include follicular pattern, dominated by extensive hyperplasia of lymphoid follicles; paracortical areas pattern, with proliferation and expansion of the paracortical areas and only a few small remnants of lymphoid follicles; diffuse pattern, characterized by diffuse hyperplasia of the paracortical areas, with no lymphoid follicular structures observed; necrotic pattern, characterized by proliferative expansion of the paracortical areas, focal scattered necrosis and nuclear fragmentation; mixed patterns of lymphoid follicles and paracortical areas; and a mixed pattern of diffuse and paracortical areas. It is also mentioned in the text that in almost all morphological patterns, moderate to severe hyperplasia of histiocytes is observed, and there are more CD8-positive T cells than CD4-positive T cells. The morphological features of lymph nodes in our case should belong to the mixed pattern of lymphoid follicles and paracortical areas, which were expanded with proliferative and atrophic lymphoid follicular structures, and a slight predominance of CD8-positive T lymphocytes.

Patients with AOSD often present with multiple enlarged lymph nodes, and when the diagnosis is still difficult to establish by a combination of clinical manifestations, laboratory tests, and imaging studies, surgical resection with pathological biopsy of lymph nodes is the inevitable choice for a definitive diagnosis. Previous studies have shown that most lymph node lesions histologically exhibit reactive hyperplasia in the paracortical region, characterized by the proliferation of immunoblasts and high endothelial venules [10, 11]. The histomorphological features of AOSD lymphadenopathy are complex and diverse and change dynamically with the course of the disease, which still needs to be differentiated from the following diseases. (1) Angioimmunoblastic T-cell lymphoma (AITL) is a T-cell lymphoma formed by the proliferation of mature follicular helper T cells with prominent hyperplasia of high endothelial venules and follicular dendritic cells, often showing generalized lymphadenopathy, hepatosplenomegaly, systemic symptoms, polyclonal hypergammaglobulinemia, and a rash with pruritus. There are many similarities between AITL and AOSD in clinical presentation and histomorphological features, with the former neoplastic cells often expressing CXCL13, PD1, CD10, BCL-6, and ICOS, most of which often show Epstein-Barr virus (EBV)-positive B cells, and an irregular proliferation of CD21-positive follicular dendritic cells surrounding high endothelial venules. (2) Dermatopathic lymphadenopathy is a special type of proliferative lesion of the paracortical region of lymph nodes that usually presents as lymphadenopathy in the drainage area with chronic skin irritation and often shows a pale nodular appearance in the paracortical region, which is mainly composed of proliferating interdigitated dendritic cells, Langerhans cells, and pigment-laden histiocytes. Some studies have shown that in dermatopathic lymphadenopathy lesions [12], the paracortical areas of lymph nodes contain at least three subsets of dendritic cells with different immunophenotypes: interdigitated dendritic cells (S-100 positive, CD1a sparsely positive, langerin negative), Langerhans cells (S-100 positive, CD1a positive, langerin positive), and few dendritic cells (S-100 positive, CD1a negative, langerin negative). (3) Infectious mononucleosis is an EBV infection-induced proliferative lesion of the lymph nodes and tonsils that is commonly observed in adolescents and young adults and has a short disease course. Its histologic features vary with disease duration. Lymphofollicular hyperplasia predominates early in the disease, with monocytoid B-cell and histiocytic hyperplasia. The later stages of the disease show proliferative expansion in the paracortical areas, comprising proliferating immunoblasts, small- to medium-sized lymphocytes, and plasma cells with a mottled appearance, dominated by CD8-positive T cells. Moreover, the immunoblasts often show Epstein-Barr virus-encoded small RNA positivity. (4) Histiocytic necrotizing lymphadenitis (Kikuchi’s disease), also known as Kikuchi-Fujimoto lymphadenitis, usually has a self-limited predilection for young adults, especially young Asian women. It is classified into three subtypes: proliferative, necrotic, and xanthomatous. The early stage is dominated by the proliferation of immunoblasts, crescentic histiocytes, and plasmacytoid dendritic cells in the paracortical region. The necrotic phase shows patchy necrosis without neutrophil infiltration in the paracortical area, with a large number of nuclear debris. The xanthoma stage contains a large number of foamy histiocytes and few immunoblasts. When AOSD lymphadenopathy appears in a necrotic pattern, it needs to be differentiated from Kikuchi’s disease; moreover, the simultaneous appearance of both lesions has also been documented [13]. (5) Systemic lupus erythematosus (SLE) is an autoimmune connective tissue disease. Up to 60% of patients have generalized or local lymphadenopathy, and the most common is involving neck and mesenteric lymph nodes. When lymph nodes are involved, it is called systemic lupus erythematosus (SLE) - associated lymphadenitis (also known as lupus lymphadenitis). Histologically, hematoxylin body and different degrees of coagulation necrosis is the characteristic morphological change of lupus lymphadenitis. Hematoxylin bodies are often seen in necrotic areas and sinuses;The necrotic area is usually large, mainly composed of lymphoid cells, abundant nuclear fragments and residual shadows of histiocytes. Sometimes there are a large number of plasma cell infiltration in germinal center and medullary cord, and neutrophil infiltration will also be encountered. Kikuchi’s disease and lupus lymphadenitis (LL) often show the same immunophenotype and have overlapping histological characteristics. Histologically, it is almost impossible to distinguish between Kikuchi’s disease and lupus lymphadenitis. Therefore, it is necessary to further integrate clinicopathological information and apply C4d immunohistochemical staining to distinguish lupus lymphadenitis from Kikuchi’s disease. Lupus lymphadenitis will show C4d deposition [14].

Depending on the course of the disease, patients with AOSD have been clinically classified into three different clinical patterns (monocyclic, multicyclic, and slowly progressive) [15, 16]. The chronic progression pattern is most commonly characterized by the occurrence of at least one persistent symptom lasting more than 1 year; it is mainly characterized by stable disease progression, persistent inflammation, and often erosion of the affected joint, followed by a multicyclic pattern, manifesting as periodic recurrences with unpredictable deterioration months or years later. A monocyclic pattern, manifesting as a single episode over 2 months but less than 1 year, persists in remission with no recurrence throughout follow-up. A new approach has divided patients with AOSD into two phenotypes: those with systemic features and those with chronic arthritis as the predominant feature [17].

AOSD often presents as a chronic passage, and patients may develop different complications within the course of the disease, which affects their clinical condition, treatment, and prognosis. Secondary hemophagocytic lymphohistiocytosis, also known as macrophage activation syndrome, is the most severe complication associated with high mortality. Its common complications include coagulopathy with multiorgan involvement, including the heart, lung, liver, spleen, and other sites [17, 18], and these patients often require more intensive treatment and have a worse prognosis. It has been shown that more than 20% of patients with AOSD experience recurrence and that patients with severe disease at the initial stage of the disease may be at an increased risk of recurrence, which requires intensive treatment and close follow-up [19].

## Data Availability

The datasets used and/or analyzed during the current study are available from the corresponding author upon reasonable request.

## Abbreviations

AOSD: Adult-onset Still’s disease
CRP: C-reactive protein
AITL: Angioimmunoblastic T-cell lymphoma
DL: Dermatopathic lymphadenopathy
IM: Infectious mononucleosis
HLH: Hemophagocytic lymphohistiocytosis
MAS: Macrophage activation syndrome
